# A phase 3 double-blind randomized (CONSORT-compliant) study of azilsartan medoxomil compared to valsartan in Chinese patients with essential hypertension: Erratum

**DOI:** 10.1097/MD.0000000000022168

**Published:** 2020-09-04

In the article, “A phase 3 double-blind randomized (CONSORT-compliant) study of azilsartan medoxomil compared to valsartan in Chinese patients with essential hypertension”,^[1]^ which appeared in Volume 99, Issue 32 of *Medicine*, Figure 5 was originally published with extra labels on the scheduled time axes (morning and afternoon/evening). This has been updated in the published version.
